# Efficacy and safety evaluation of bright light therapy in patients with post-stroke insomnia: A protocol for systematic review and meta-analysis

**DOI:** 10.1097/MD.0000000000027937

**Published:** 2021-12-17

**Authors:** Huabin Lei, Wei Wang, Yinan Cao, Yaru Ma, Xusheng Xue

**Affiliations:** Sun Simiao Hospital, Beijing University of Chinese Medicine, Tongchuan, Shaanxi, China.

**Keywords:** bright light therapy, post-stroke insomnia, protocol, systematic review

## Abstract

**Background:**

Post-stroke insomnia (PSI) is a common and severe illness among the complications of stroke. Although there are plenty of drugs currently used for PSI treatment, they generate several side effects and other problems. Bright light therapy (BLT) is thought to be relatively safe and effective in treating PSI patients. Despite this, there is still a lack of systematic review on BLT in the treatment of PSI. Allowing for this, the aim of this study is to assess the efficacy and safety of BLT for PSI.

**Methods:**

The meta-analysis and systematic review will perform a comprehensive electronic search for items fulfilling the required criteria in Web of Science, Google Scholar, Wan Fang database, MEDLINE, Baidu Scholar, PubMed, SinoMed, Embase, Chinese Biomedical Literature Database (CBM), China national knowledge infrastructure database (CNKI), Cochrane Library Central Register of Controlled Trials (CENTRAL), and Wei Pu database from establishment to January 1, 2022. We will select articles, collect data, and assess the methodology quality. And we will set the primary outcome and secondary outcomes in this research. RevMan 5.3 software will be used to analyze the data for this investigation.

**Results:**

The work of this research will be published in peer-reviewed scientific journals.

**Conclusion:**

The aim of this study is to assess the efficacy and safety of BLT for PSI and present robust scientific evidence concerning BLT for PSI.

**Registration:**

INPLASY2021100065.

## 1 Introduction

As one of the acute cerebrovascular diseases, stroke is a main cause of death and disability globally, and presents a high risk of recurrence.^[1]^ The typical feature of stroke is sleep disorders, and the most common type of sleep disorders is insomnia.^[2]^ Currently, insomnia is a condition primarily characterized by trouble falling asleep, difficulties maintaining sleep, or experiencing sleep as non-restorative.^[3]^ When left untreated, it may even lead to such problems as depression, cognitive impairment, or other diseases.^[4]^ And it has been reported that insomnia may aggravate the risk of stroke.^[5]^ Moreover, substantial evidence has implicated that poor sleep is a high risk factor for stroke and lead to aggravation of the disease state.^[6,7]^ Therefore, it is especially important to effectively improve sleep quality in patients with PSI.

At present, treatment of PSI mainly includes pharmacological therapy and non-pharmacological therapy.^[8]^ Different sorts of medications are used in insomnia pharmacotherapy.^[9]^ Most sleeping pills, such as most tranquilizers, come from drugs called benzodiazepines. Such drugs include sedatives, such as Valium, chlordiazepoxide, nitrazepam, etc.^[10]^ Nevertheless, researches demonstrated that the long-term administration of benzodiazepines and other hypnotic drugs may cause severe side effects such as dependence, vertigo, cognitive impairment, and so on.^[11–14]^ On the contrary, PSI patients usually present motor and cognitive impairment and insomnia has long been treated with nonpharmacological methods.^[15]^ Although nonpharmacological intervention such as education on sleep hygiene and exercise is recommended for PSI patients, these treatments can be burdensome for some PSI patients.^[11,16]^ Therefore, it is necessary to explore new therapeutic modalities that are safe, effective, and simple for PSI patients.

Bright light therapy (BLT) is a natural, simple, and low-cost treatment that does not cause patients any problems with lingering effects or tolerance. Typically, patients are instructed to be exposed to bright light at a constant distance every day.^[12,17]^ The effects of BLT are crucial in promoting serotonin secretion and inhibits melatonin secretion, which has beneficial effects on sleep quality.^[11,16]^

BLT has been shown in prior researches to improve the quality of sleep in stroke patients.^[16–19]^ To date, however, there has not been a systematic review of the efficacy and safety of BLT for PSI, and the evidence of BLT for PSI is unclear and need to be further studied. Hence, the objective of this study is to perform the study to determine the clinical efficacy and safety of BLT for PSI, and provide a valuable basis of BLT for PSI.

## 2 Methods

### 2.1 Protocol registration

The content of this project follows the guidance of the preferred reporting items for systematic reviews and meta-analyses protocols (PRISMA-P). And our protocol was registered with the International Platform of Registered Systematic Review and Meta-Analysis Protocols (INPLASY) on 18 October 18, 2021. And our protocol was last updated on November 1, 2021. INPLASY registration number of this study is INPLASY2021100065.

### 2.2 Inclusion and exclusion criteria

#### 2.2.1 Types of studies

Randomized controlled trials (RCTs) will be used to investigate the efficacy and safety of BLT for PSI. And there will be no language, publication date, or publication status restrictions. Moreover, we will exclude non-RCTs, cross-experimental studies, the experience of expert, case studies, researches of laboratory animals, reviews, and duplicate documents. In addition, studies with ambiguous designs or inadequate data will be removed.

#### 2.2.2 Research objects

People with a diagnosis of PSI will be considered for inclusion, regardless of their gender, age, race, the extent of disease, family status, household wealth, or educational attainment. In the meantime, PSI patients who are pregnant or nursing women will be excluded. In addition, we will exclude PSI patients with other severe illnesses, including cancer, heart, liver, or lung disease.

#### 2.2.3 Types of intervention

In the intervention groups, intervention measures will be BLT alone or BLT as the major part of the combination therapy. And there will be no restrictions applied to the frequency, duration, location, and color of BLT. In the control groups, intervention measures will include no treatment, sham treatment (such as sham light, dim red light, or negative ion), or placebo.

#### 2.2.4 Outcomes selection

On the one hand, we will base the Pittsburgh Sleep Quality Index to obtain the overall efficacy as the primary outcomes. On the other hand, the secondary outcomes of the study will be the following three items: Recurrence rate, rate of adverse reactions, and quality of life calculated by Quality of Life Scale (SF-36).

### 2.3 Search strategy

Until January 1, 2022, we shall do thorough searches without language restrictions in the following databases using medical subject headings and keywords related with BLT for PSI: Web of Science, Google Scholar, Wan Fang database, MEDLINE, Baidu Scholar, PubMed, SinoMed, Embase, Chinese Biomedical Literature Database (CBM), China national knowledge infrastructure database (CNKI), Cochrane Library Central Register of Controlled Trials (CENTRAL), and Wei Pu database. PubMed's search method is summarized in Table 1. Analogously, we will set the strategies with a slight adjustment for searches in the other websites.

**Table 1:** Search strategy in PubMed.

Number	Entry terms
#1	“Stroke” [MeSH Terms] or “Stroke” [Title/Abstract] or “Strokes” [Title/Abstract] or “Cerebrovascular Accident” [Title/Abstract] or “Cerebrovascular Accidents” [Title/Abstract] or “CVA (Cerebrovascular Accident)” [Title/Abstract] or “CVAs (Cerebrovascular Accident)” [Title/Abstract] or “Cerebrovascular Apoplexy” [Title/Abstract] or “Apoplexy, Cerebrovascular” [Title/Abstract] or “Vascular Accident, Brain” [Title/Abstract] or “Brain Vascular Accident” [Title/Abstract] or “Brain Vascular Accidents” [Title/Abstract] or “Vascular Accidents, Brain” [Title/Abstract] or “Cerebrovascular Stroke” [Title/Abstract] or “Cerebrovascular Strokes” [Title/Abstract] or “Stroke, Cerebrovascular” [Title/Abstract] or “Strokes, Cerebrovascular” [Title/Abstract] or “Apoplexy” [Title/Abstract] or “Cerebral Stroke” [Title/Abstract] or “Cerebral Strokes” [Title/Abstract] or “Stroke, Cerebral” [Title/Abstract] or “Strokes, Cerebral” [Title/Abstract] or “Stroke, Acute” [Title/Abstract] or “Acute Stroke” [Title/Abstract] or “Acute Strokes” [Title/Abstract] or “Strokes, Acute” [Title/Abstract] or “Cerebrovascular Accident, Acute” [Title/Abstract] or “Acute Cerebrovascular Accident” [Title/Abstract] or “Acute Cerebrovascular Accidents” [Title/Abstract] or “Cerebrovascular Accidents, Acute” [Title/Abstract]
#2	“Sleep Initiation and Maintenance Disorders” [MeSH Terms] or “Sleep Initiation and Maintenance Disorders” [Title/Abstract] or “Disorders of Initiating and Maintaining Sleep” [Title/Abstract] or “DIMS (Disorders of Initiating and Maintaining Sleep)” [Title/Abstract] or “Early Awakening” [Title/Abstract] or “Awakening, Early” [Title/Abstract] or “Nonorganic Insomnia” [Title/Abstract] or “Insomnia, Nonorganic” [Title/Abstract] or “Primary Insomnia” [Title/Abstract] or “Insomnia, Primary” [Title/Abstract] or “Transient Insomnia” [Title/Abstract] or “Insomnia, Transient” [Title/Abstract] or “Rebound Insomnia” [Title/Abstract] or “Insomnia, Rebound” [Title/Abstract] or “Secondary Insomnia” [Title/Abstract] or “Insomnia, Secondary” [Title/Abstract] or “Sleep Initiation Dysfunction” [Title/Abstract] or “Dysfunction, Sleep Initiation” [Title/Abstract] or “Dysfunctions, Sleep Initiation” [Title/Abstract] or “Sleep Initiation Dysfunctions” [Title/Abstract] or “Sleeplessness” [Title/Abstract] or “Insomnia Disorder” [Title/Abstract] or “Insomnia Disorders” [Title/Abstract] or “Insomnia” [Title/Abstract] or “Insomnias” [Title/Abstract] or “Chronic Insomnia” [Title/Abstract] or “Insomnia, Chronic” [Title/Abstract] or “Psychophysiological Insomnia” [Title/Abstract] or “Insomnia, Psychophysiological” [Title/Abstract]
#3	“Phototherapy” [MeSH Terms] or “Phototherapy” [Title/Abstract] or “Phototherapies” [Title/Abstract] or “Therapy, Photoradiation” [Title/Abstract] or “Photoradiation Therapies” [Title/Abstract] or “Therapies, Photoradiation” [Title/Abstract] or “Light Therapy” [Title/Abstract] or “Light Therapies” [Title/Abstract] or “Therapies, Light” [Title/Abstract] or “Therapy, Light” [Title/Abstract] or “Photoradiation Therapy” [Title/Abstract]
#4	“Randomized controlled trial” [Publication Type] or “Randomized controlled trial” [Title/Abstract]
#5	#1 and #2 and #3 and #4

### 2.4 Data collection and management

#### 2.4.1 Literature screening

**Figure 1:**
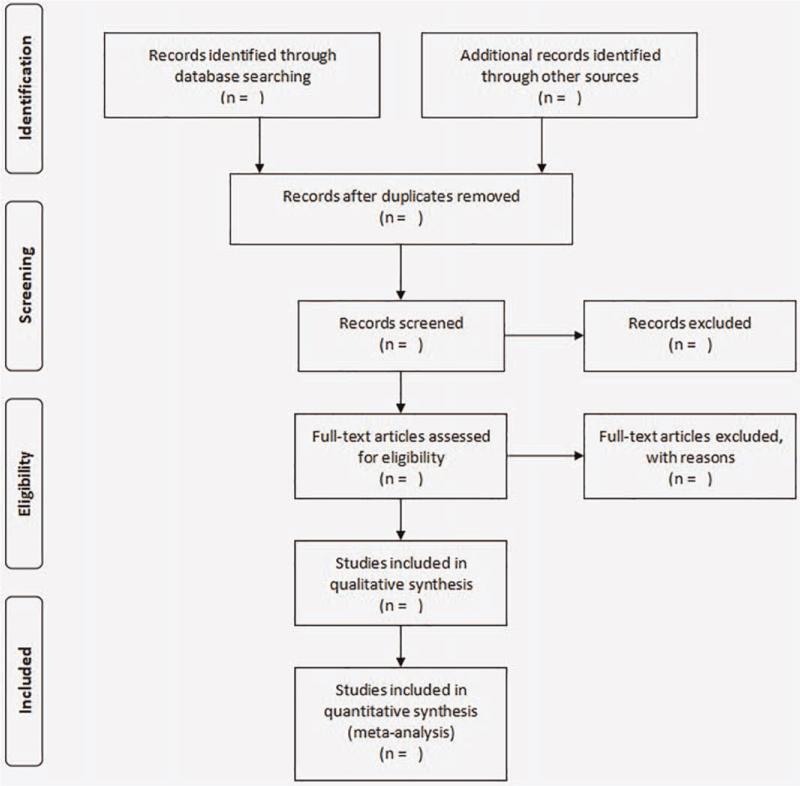
Flow diagram of the study selection process.

First, the initial literature will be searched and imported into EndnoteX9 using the study's search strategies. Second, after importing citations into EndnoteX9 tool, duplicates will be removed. After that, 2 authors (LHB and WW) will independently scan and screen the titles and abstracts of literature identified through the search strategy to exclude literature that does not meet the requirements of this study. Then, 2 authors (LHB and WW) will read the eligible papers in their entirety in order to determine their eligibility. In the event that the 2 authors (LHB and WW) disagree, a consensus will be reached by discussing and consulting with a third author (XXS). The process of literature screening is presented using a flow chart as shown in Fig. 1.

#### 2.4.2 Data extraction

Two independent researchers (LHB and WW) will be responsible for refining the data from the selected studies. The following information will be extracted: general literature information, study methodologies, all participant information, intervention and control information, outcome indexes, adverse reactions, etc. We shall endeavor to contact the corresponding author in the included publications, or consult a third author (XXS), or conduct internal in-depth discussions if the data is missing, erroneous, or ambiguous.

#### 2.4.3 Assessment of risk of bias

Two authors (CYN and XXS) will objectively perform the risk of bias assessment using the Cochrane risk-of-bias tool in the included RCTs. And they will consider the following aspects to determine the bias: random sequence generation, the concealment of allocation, blinding of participants and personnel, blinding of result evaluation, the integrity of the report data, selective reporting of outcomes, other sources of bias. And each study will be assigned to 1 of 3 categories: “Low risk,” “High risk,” or “Unclear risk.”^[20]^ Furthermore, if there is any disagreement, we can contact the corresponding author in the publications we have collected, or consult the third author (WW), or conduct detailed discussions to solve it.

#### 2.4.4 Measures of treatment efficacy

One the one hand, for continuous data, the efficacy measure will be mean differences (MD) or standard mean differences (SMD) with 95% confidence intervals (CIs). On the other hand, for the description of dichotomous data, we will use relative risk (RR) to represent the effect size.

#### 2.4.5 Dealing with missing data

If some data are absent, we must try our best to contact the first author or correspondent author by e-mail or phone in order to collect the needed information. On condition that complete information cannot be supplied or unable to contact the author, we will run a limited analysis based on the available data and examine the potential impact of missing data in the discussion.^[8]^

#### 2.4.6 Determination of heterogeneity

The *I*^2^ statistic and the chi-squared test will be used to evaluate the criteria for heterogeneity. *I*^2^ ≥ 50% will be considered to indicate substantial heterogeneity. Conversely, *I*^2^ < 50% will represent a low level of heterogeneity. At the same time, if there is significant heterogeneity, a subgroup analysis or a sensitivity analysis will be used to investigate possible explanations.

#### 2.4.7 Assessment of publication bias

If more than 10 RCTs are available to us, we will need to draw funnel plots and performed Egger test to investigate possible publication bias.

#### 2.4.8 Data synthesis and analysis

The data will be analyzed by RevMan 5.3 software (The Cochrane Collaboration, Oxford, UK). The fixed-effects approach will be applied for data synthesis with a low level of heterogeneity (*I*^2^ < 50%). Also, if heterogeneity is substantial *(I*^2^ ≥ 50%), the random-effect approach will be applied for data synthesis. Besides, if it is unable to judge and test the source of heterogeneity, the narrative synthesis analysis will be carried out.

#### 2.4.9 Subgroup analysis

We will need to do a subgroup analysis when necessary to investigate plausible sources of heterogeneity based on interventions, participant information, and outcome indexes.

#### 2.4.10 Sensitivity analysis

If there is a large degree of heterogeneity in the analysis, we will perform a sensitivity analysis to identify the quality and stability of the conclusions by excluding the low-quality studies.

#### 2.4.11 Quality of evidence

All outcomes’ evidence quality will be evaluated according to the Grading of Recommendations Assessment, Development, and Evaluation (GRADE) working group approach. The quality of evidence will be graded as high, moderate, low, or very low. In addition, the strength of evidence recommendation will be graded as strong or weak.

### 2.5 Ethics and publication

As there is no individual data from participants in this study, it will not need ethics approval. Furthermore, we will publish this research, which evaluates the efficacy and safety of BLT in patients with PSI, in a peer-reviewed scientific journal.

## 3 Discussion

PSI is a complication associated with stroke, and it is one of the most frequently occurring types of sleep disorders. It has been reported that 37% to 59% of stroke patients suffer from insomnia.^[7]^ Insomnia often causes functional impairment and accelerates the development of other physical and mental illnesses.^[21]^ In addition, insomnia consumes a lot of economic and medical expenses. Evidence suggests that insomnia PSI is one of the major risk factors for stroke recurrence, and it might be directly associated with stroke mortality.^[22]^ In addition, if the condition of PSI is not treated properly in a timely manner, insomnia could even lead to suicidal ideation in severe cases.^[23]^ In recent years, despite there are more and more studies investigating the treatment of PSI, the treatment for PSI has remained limited.

Given the side effects and the high cost of drug treatments for PSI, pharmacological therapy still needs to be improved.^[11,12]^ Meanwhile, many limitations still affect the interventions of education on sleep hygiene and exercise.^[16]^ Therefore, it is crucial to improve the treatment modalities for PSI patients, and clinicians need to adopt more aggressive treatment for PSI patients.

Light is a critical factor in the control of sleep and wakefulness, and it has been used in the clinical treatment of sleep disorders since the 1970 s.^[24]^ BLT has been demonstrated to be a beneficial treatment for PSI patients in earlier trials and it is less likely to cause side effects. Despite this, there are yet no systematic reviews on the efficacy and safety of BLT for PSI that have been published. We sincerely hope that the study would provide additional references to help clinicians with PSI. However, our research still has several potential limitations and disadvantages. First, there may be substantial heterogeneity due to the quality of different studies. Next, due to different methods of BLT, heterogeneity may be higher. Finally, some RCTs may be of poor quality and there may be a potential risk of bias.

## Author contributions

**Data curation:** Huabin Lei, Wei Wang, Xusheng Xue

**Formal analysis:** Wei Wang, Yinan Cao, Xusheng Xue

**Investigation:** Yinan Cao, Yaru Ma.

**Methodology:** Wei Wang, Xusheng Xue, Yaru Ma

**Project administration:** Huabin Lei, Xusheng Xue, Yinan Cao, Yaru Ma

**Resources:** Yinan Cao, Yaru Ma, Wei Wang, Yinan Cao

**Software:** Wei Wang, Yinan Cao, Xusheng Xue, Yaru Ma

**Visualization:** Huabin Lei, Yaru Ma, Xusheng Xue

**Writing – original draft:** Huabin Lei, Wei Wang, Yaru Ma

**Writing – review & editing:** Yinan Cao, Xusheng Xue
